# (Na, Zr) and (Ca, Zr) Phosphate-Molybdates and Phosphate-Tungstates: I–Synthesis, Sintering and Characterization

**DOI:** 10.3390/ma16030990

**Published:** 2023-01-20

**Authors:** M. E. Karaeva, D. O. Savinykh, A. I. Orlova, S. A. Khainakov, A. V. Nokhrin, M. S. Boldin, S. Garcia-Granda, A. A. Murashov, V. N. Chuvil’deev, P. A. Yunin, A. A. Nazarov, N. Y. Tabachkova

**Affiliations:** 1 Materials Science Department, Physical and Technical Research Institute, Lobachevsky State University of Nizhny Novgorod, 603022 Nizhny Novgorod, Russia; 2 Faculty of Chemistry, University of Oviedo, 33006 Oviedo, Spain; 3 Laboratory of Diagnostics of Radiation Defects in Solid State Nanostructure, Institute for Physics of Microstructure, Russian Academy of Science, 603950 Nizhniy Novgorod, Russia; 4 Center Collective Use “Materials Science and Metallurgy”, National University of Science and Technology “MISIS”, 119991 Moscow, Russia; 5 Laboratory “FIANIT”, Laser Materials and Technology Research Center, A.M. Prokhorov General Physics Institute of the Russian Academy of Sciences, 119991 Moscow, Russia

**Keywords:** NZP, ceramics, spark plasma sintering, thermal expansion

## Abstract

Submicron-grade powders of Na_1-x_Zr_2_(PO_4_)_3-x_(XO_4_)_x_ compounds (hereafter referred to as NZP) and Ca_1-x_Zr_2_(PO_4_)_3-x_(XO_4_)_x_ compounds (hereafter, CZP), X = Mo, W (0 ≤ x ≤ 0.5) were obtained by sol-gel synthesis. The compounds obtained were studied by X-ray diffraction phase analysis and electron microscopy. An increase in the W or Mo contents was shown to result in an increase in the unit cell volume of the NZP and CZP crystal lattices and in a decrease in the coherent scattering region sizes. Thermal expansion behavior at high temperatures of synthesized NZP and CZP compounds has been investigated. The dependencies of the parameters *a* and *c* on the heating temperature, as well as the temperature dependence of the crystal lattice unit cell volume *V* in the range from the room temperature up to 800 °C, were obtained. The dependencies of the average thermal expansion coefficient (α*_av_*) and of the volume coefficient (β) on the W and Mo contents in the compositions of NZP and CZP compounds were studied. Ceramics Na_1-x_Zr_2_(PO_4_)_3-x_(XO_4_)_x_ with relatively high density (more than 97.5%) were produced by spark plasma sintering (SPS). The increase in the W or Mo contents in the ceramics leads to an increase in the relative density of NZP and to a decrease of the optimum sintering temperature. The mean grain size in the NZP ceramics decreases with increasing W or Mo contents. The study of strength characteristics has revealed that the hardness of the NZP ceramics is greater than 5 GPa, and that the minimum fracture toughness factor was 1 MPa·m^1/2^.

## 1. Introduction

In this paper, the inorganic compounds with the NaZr_2_(PO_4_)_3_ (NZP-type) structure are studied extensively in connection with the prospects of application for solving the problems of immobilization of the highly active components of radioactive waste (HLW) [1,2,3,4]. In particular, the Mo-containing fractions of the radioactive waste solidify as inclusions in glass, together with other nuclides, for example, the wedge-shaped glass ceramics in the borosilicate glasses [5,6,7]. When more than 10 wt.% of Mo is incorporated into a multi-component highly radioactive waste, Mo can form powellite—a mineral of Ca molybdate, CaMoO_4_. This affects the chemical resistance of the glasses, adversely affecting the solubility of molybdates [7]. A similar situation takes place with W.

Ceramics with an NZP-type structure can be quite effective at binding W and Mo into stable crystalline compounds where W and Mo can partially replace P [8,9,10,11,12,13,14,15,16]. Stable crystalline NZP-like matrix substances are most suitable for binding Mo and W. The inclusion of Mo in NZP-like ceramics leads to a decrease in the Mo leaching rate as compared with the glasses, glass ceramics, and Synroc materials containing individual phases of readily soluble molybdates [8]. The sintering of the crystalline compounds into ceramics increases their relative density, while preserving the phase composition. At the same time, the resulting ceramics have high strength characteristics [1,4,17,18,19,20].

The compounds with the NaZr_2_(PO_4_)_3_ structure are characterized by the crystal chemical formula (M1)^VI^(M2)^VIII^_3_[L^VI^_2_(XO_4_)_3_]^n-^,here M1 and M2 are the sites in the voids of the [L^VI^_2_(XO_4_)_3_]^n-^ framework. The family of phosphates with the NZP structure includes many compounds, due to the possibility of isomorphic substitutions in various positions of the structure [21,22,23,24,25,26,27,28,29,30,31,32,33]. The framework of the structure is formed by multiply charged cations L with oxidation states 5+, 4+, 3+, or 2+ of small sizes and by anions XO^4-^. Most compounds of the NZP family contain P as an anion-forming element X. However, there are also compounds with the NZP-type structure, in which P is replaced by other anions. Some compounds are known where P is replaced by Si [17,24,25,26,27], by S [28,29], by V [30], by As [31], by Se [32], and by Mo [8,9,33].

The M sites can be occupied fully or partially, or remain vacant. The composition of NZP-type phosphates can include cations in the oxidation states from +1 to +4; mainly, low-charged and relatively large cations occur. The presence of four positions (M1, M2, L, and X) capable of incorporating cations of various sizes determines the prospects for the application of materials based on compounds with the NZP-type structure in various fields. The compounds of this structure are characterized by high ionic conductivity as well as by high corrosion, thermal, radiation, and chemical resistance, along with high catalytic activity [33,34].

The behavior of phosphates under heating and the values of thermal expansion coefficients depend on the charge, size, and electronegativity of their constituent ions. Due to the ability of the structure to accommodate various constituent elements, it becomes possible to develop various materials with desired thermal expansion coefficients [35]. In most cases, these compounds are characterized by expansion of the unit cell along the *c* crystallographic axis and by compression along the *a* and *b* axes when heated (anisotropic thermal expansion). In the case of a partial or complete substitution of the anions, the charge (*n*) of the framework changes. In this case, the cations in the Msites compensate for this charge, preserving the electroneutrality. Therefore, the cations in the M sites and the total occupancy of the sites affect the changes in the thermal expansion coefficients. Some of these compounds have small or ultra-low [down to (1–2)·10^−6^ deg^−1^] controlled thermal expansion coefficients [1,4,36,37,38,39,40,41,42,43]. Also, these compounds are stable under hydrothermal conditions at temperatures up to 400 °C and at durations of contact with water up to two years [1,4,18,44,45,46]. Such a unique hydrolytic resistance of the ceramics with the NZP-type structure increases interest in them as materials for solving the radiochemical problems of immobilization of radioactive waste [1,4,21].

At present, hot pressing or conventional sintering of preliminary pressed powders are applied for sintering the mineral-like ceramics most often [18,19,20,47]. The necessity of maintaining elevated sintering temperatures for long periods to provide a high density of the ceramics is the drawback of these methods. Spark plasma sintering (SPS) stands out from other methods of obtaining the ceramic samples. The method consists of high-speed heating (up to 2500 °C/min) of a powder material in vacuum by passing a series of short electric current pulses through a sample and a mold, while applying pressure [48,49,50,51,52]. This method is characterized by high shrinkage rates at reduced sintering temperatures. The sintering of the mineral-like ceramic samples up to high relative densities (about 95%) takes place in shorter times [27,51,52,53]. This ensures the reduction of the times of handling the highly active components of the radioactive waste as compared to hot pressing methods and conventional sintering of the powders pressed in advance. These advantages of SPS allow a significant expansion of applications in the development of ceramic matrices for the immobilization of high-level waste (HLW) and in the transmutation of minor actinides [4,27,51,52,53,54,55,56].

Ceramic materials obtained by SPS are characterized by a high relative density and enhanced physical and mechanical properties, which open up new possibilities for obtaining the ceramic materials for various purposes [4,27,48,49,50,51,52]. Some of these ceramics have a high radiation resistance [4,52,53,57] and high hydrolytic stability [4,56,58,59].

This work is devoted to the synthesis and investigation of properties of the NZP-framework orthophosphates with partial substitution of the P ions by Mo and W and various cations in the structure voids. These ceramics may potentially be applied to the immobilization of the Mo- and W-containing fractions of radioactive waste.

## 2. Materials and Methods

Solid solutions of the Na_1-x_Zr_2_(PO_4_)_3-x_(XO_4_)_x_ and Ca_1-x_Zr_2_(PO_4_)_3-x_(XO_4_)_x_ types, where X = Mo, W, and x = 0.1, 0.2, 0.3, 0.4, 0.5, were chosen for investigation. The compounds were synthesized by the sol-gel method. A sample of Zr oxychloride was dissolved in a mixture of solutions of ammonium molybdate or tungstate and Na or Ca nitrate. A 1 M solution of ammonium dihydrogen phosphate was added dropwise to the resulting solution in the course of continuous stirring. As a result, gel-like precipitates were formed. The reagents were mixed in the stoichiometric proportions. For more complete precipitation, a salting-out agent (ethyl alcohol) was added. After brief stirring, the gel was dried at 90 °C for 1 day. The gel-like sediment was dried in a drying cabinet and then was dispersed in an agate mortar, heated up to 600 °C, dispersed again, and heated up to 800 °C. The resulting powders were investigated by X-ray diffraction (XRD) between the stages of annealing.

**Figure 1:**
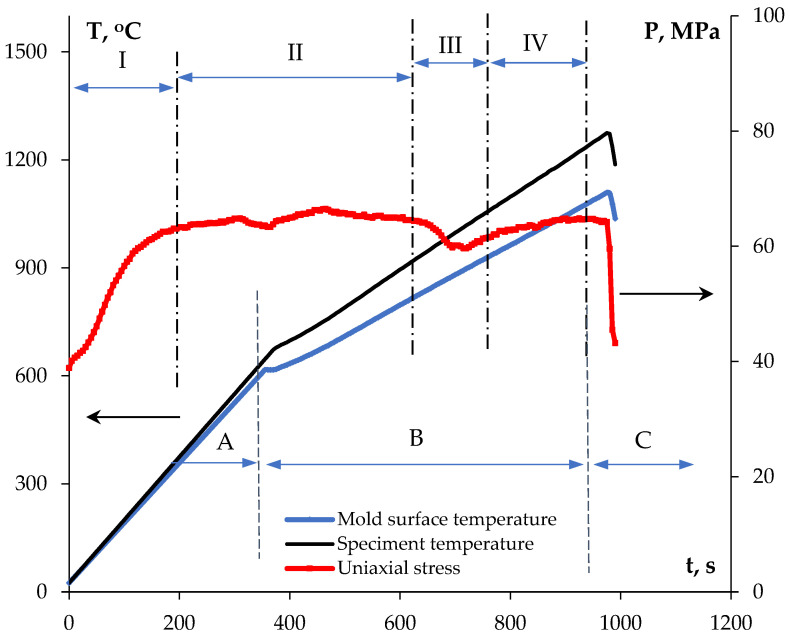
Regular sintering diagram “temperature-stress-time” for SPS. Na_0.7_Zr_2_(PO_4_)_2.7_(MoO_4_)_0.3_ compound.

The ceramics were sintered from the obtained powders by SPS using Dr. Sinter™ model SPS-625 (SPS SYNTEX^®^, Kanagawa, Japan). Prior to sintering, the powders were ground in an agate mortar for 5 min, and pressed into a graphite mold at a pressure of 70 MPa. No binders were used for the preparation of the initial powder green body. The binderless powders were placed in a graphite mold with an inner diameter of 12.8 mm and an outer diameter of 30 mm, and heated up by passing millisecond pulses of high-power direct electric current (up to 5 kA). Sintering was performed in a vacuum. The effective temperature (T_1_) was measured with a Chino IR-AHS2 pyrometer (Chino Corporation, Tokyo, Japan) focused on the graphite mold surface. Based on previous studies, and on the comparison of the infrared pyrometer readings (T_1_ in Figure 1) to those of a thermocouple attached to the ceramic specimen surface, the values of T1 were used to calculate the actual specimen temperature (T) using the following empirical relation: T = A·T_1_ − B, where A and B are empirical constants which depend on the heating rate V_h_ (Figure 1 The method of comparing temperatures T and T_1_ is described in [60]. To improve the contact of the powder sample with the graphite mold and to compensate for the difference in the thermal expansion coefficients of graphite and the ceramics, a graphite foil was placed inside the graphite mold. A two-stage heating regime was used—stage A: heating up to 570–580 °C with the heating rate V_h1_ = 100 °C/min, and stage B: heating up to the sintering temperature T_s_ with the heating rate V_h2_ = 50 °C/min. Holding at the sintering temperature T_s_ was absent (t_s_ = 0 s). The average uniaxial stress applied was ~65 MPa. In order to avoid cracking of the ceramic specimen, multistage pressure regulation was applied during sintering: (stage I) a gradual increasing of the uniaxial stress P from 35–38 MPa up to 64–65 MPa during the rapid heating stage up to 570–580 °C, (stage II) holding at 64–66 MPa at 580–840 °C, (stage III) reducing the pressure to 60 MPa at 900 °C, and (stage IV) gradually increasing the pressure again to ~65 MPa to the moment of the end of shrinkage. The typical scheme of the stress and temperature regulation during SPS is presented in Figure 1. The samples were cooled down together with the setup (stage C, Figure 1). As a result of sintering, dense ceramic specimens without macrodefects were obtained.

**Figure 2:**
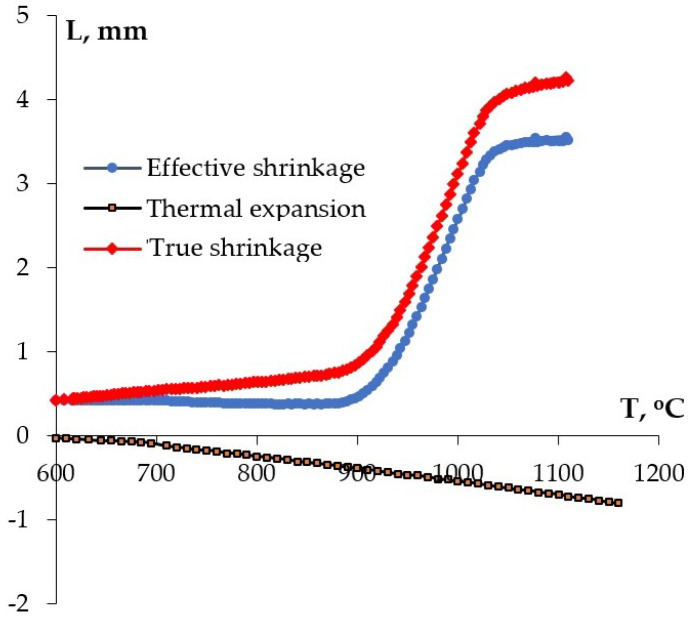
Temperature curves of the shrinkage of Na_0.7_Zr_2_(PO_4_)_2.7_(MoO_4_)_0.3_ powders: L_eff_—the effective shrinkage, L_0_—the contribution of the thermal expansion of the setup, L—true shrinkage.

The temperature curves of the effective shrinkage (L_eff_) were measured during sintering using the built-in dilatometer of the SPS-625. To account for thermal expansion of the mold (L_0_), a separate series of experiments on heating up an empty mold was carried out. True shrinkage was calculated as L = L_eff_ – L_0_ (Figure 2). Using the dependencies L(T), the temperature dependencies of the shrinkage rate were calculated: S = ΔL/Δt.

The optimal sintering temperature T_s_ for each ceramic was selected on the basis of analysis of the dependencies L_eff_(T). The samples were heated up to the temperature corresponding to the end of the stage of intensive powder shrinkage. Due to the contribution of the thermal expansion L_0_(T) on the L_eff_(T) curve, stage III arose in the temperature dependence of true shrinkage L(T), within which true shrinkage of the ceramics almost didn’t change (Figure 2). The change in temperature during stage III was ~50 °C, corresponding to the time interval of ~1 min at the heating rate V_h2_ = 50 °C/min. In the course of heating, we tried to minimize the duration of stage III, since intensive grain growth takes place in the ceramics at high temperatures (T > T_s_). The coarse-grain ceramics have very low resistance against thermal shock, and the samples are often destroyed during cooling down. Additionally, the sintering at high temperatures can lead to partial dissociation of the elements (W, Mo) from the ceramic surfaces and to more intensive saturation of the ceramic sample surfaces with the mold (see [50,61,62,63,64,65,66,67]).

The XRD phase analysis of the ceramics was performed using Bruker^®^ D8 Discover™ X-ray diffractometer (Bruker Corp., Billerica, MA, USA) in symmetric Bragg-Brentano geometry. An X-ray tube with a Cu cathode (CuK_α_ radiation) was used. The goniometer radius was 30 cm. All experiments were performed in the same conditions. The specimens, comprising cylindrical tablets of 12 mm diameter and 3 mm height, were placed on the goniometer table. At the beginning of each experiment, adjustment of the specimen height by the beam splitting method was performed with 0.1 mm slits both on the primary beam and in front of the detector. When acquiring the XRD curves, the primary beam size was limited in the equatorial plane by a 0.6 mm slit, and in the axial plane by a 12 mm slit. The XRD curves were acquired in the θ/2θ-scan mode in the angle range from 10° to 70° in 2θ. The step in angle was 0.1°. LynxEYE linear position-sensitive detector with 192 independent acquisition channels and a 2° angle aperture in 2θ was used. The dwell time was 2 s, which was equivalent to the effective dwell time about 40 s due to the use of the position-sensitive detector. The thermal expansion in the temperature range 25–800 °C was investigated using Panalytical^®^ X’Pert Pro™ high-temperature X-ray diffractometer (Malven PANAlytical B.V., Almelo, The Netherlands) with Anton Paar^®^ HTK–1200N high-temperature chamber (Anton Paar GmnH, Graz, Austria) with a step of 100 °C. The unit cell parameters were calculated with the Rietveld method using Topas 4.2 software.

The microstructure of the powders and ceramics was investigated using Jeol^®^ JSM-6490 scanning electron microscope (SEM, Jeol Ltd., Tokyo, Japan) and Jeol JEM-2100 transmission electron microscope (TEM, Jeol Ltd., Tokyo, Japan). The mean particle size (D_P_), the size of agglomerates (D_A_), and grain size (d) were calculated by the section method using GoodGrains 2.0 software (UNN, Nizhny Novgorod, Russia). To determine the mean particle sizes, investigations of the sizes of all particles of the powders presented in the images obtained by TEM were conducted. When determining the mean grain sizes, we measured 100 or more grains presented in the images obtained by SEM. When determining D_P_ and d, three photographs (images) or more were analyzed. The mean sizes were calculated with GoodGrains 2.0 software in the automated mode, on the base of analysis of the histograms of the size distributions of the particles/grains. The uncertainties of determining D_P_ and d were calculated as the standard deviations.

The density of the obtained ceramics was measured by hydrostatic weighing in distilled water using Sartorius^®^ CPA 225D balance (Sartorius AG, Göttingen, Germany). The uncertainty of the density measurement was ±0.001 g/cm^3^. The theoretical density of the ceramics (ρ_th_) was calculated on the basis of the XRD investigations. The microhardness (H_v_) of the ceramics was measured using Duramin^®^ Struers-5 hardness tester (Struers LLC, Cleveland, OH, USA). The load was 200 g. The minimum fracture toughness factor (K_IC_) was calculated according to the Palmquist method from the length of the largest radial crack forming by the Vickers pyramid during the indentation of the ceramics:

K_IC_ = 0.016(*P*/*c*^3/2^)(E/H_v_)^1/2^, (1)

where *P* is the indenter load [g], *c* is the mean distance from the imprint center to the end of the crack, and E is the elastic modulus of the material [GPa]. When calculating the magnitude of K_IC_, we used the lengths of 10 or more of the longest cracks from 10 different indents formed on the ceramic sample surfaces when loading by Vickers pyramid.

## 3. Results and Discussion

### 3.1. Synthesis and Characterization of the Powders

**Figure 3:**
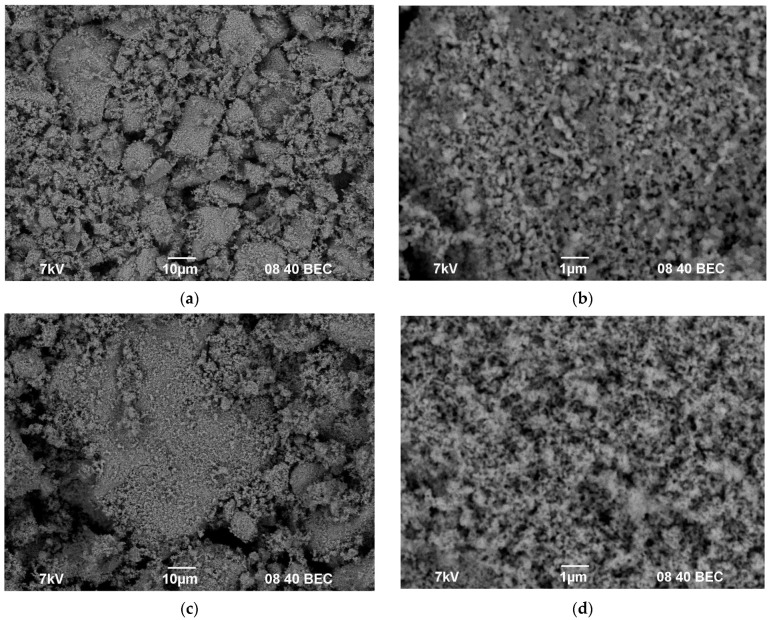

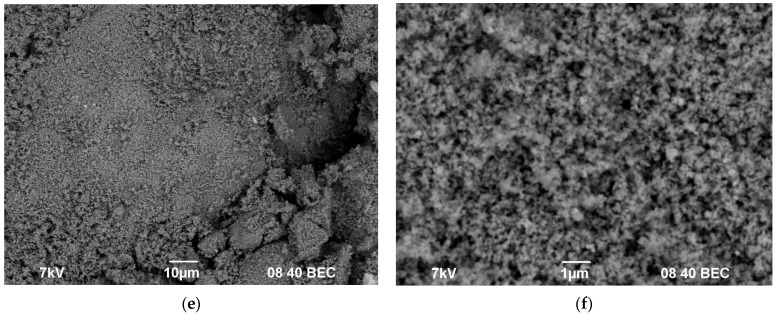
SEM images of the agglomerates (**a**,**c**,**e**) and of the synthesized powders (**b**,**d**,**f**): Ca_0.4_Zr_2_(MoO_4_)_0.1_(PO_4_)_2.9_ (**a**,**b**), Na_0.5_Zr_2_ (MoO_4_)_0.5_(PO_4_)_2_ (**c**,**d**), Na_0.5_Zr_2_ (WO_4_)_0.5_(PO_4_)_2_ (**e**,**f**).

The powder samples were light-colored. According to the SEM results, the submicron-grained powders were agglomerated; there were some large agglomerations up to 10–20 μm in size in the Ca_1-x_Zr_2_(PO_4_)_3-x_(XO_4_)_x_ powders (denoted as “CZP” in work) (Figure 3a) and ~50 μm in size in the Na_1-x_Zr_2_(PO_4_)_3-x_(XO_4_)_x_ powders (denoted as “NZP”) (Figure 3c,e) after grinding in the agate mortar. No considerable effect of the W and Mo contents on the granulometric composition and morphology of the powders was observed.

**Figure 4:**
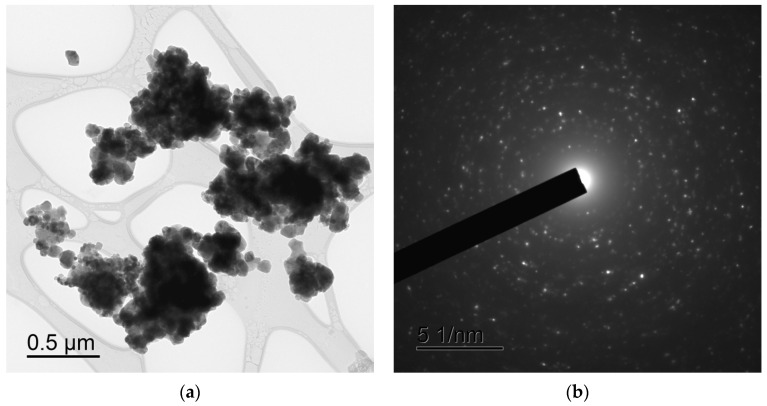

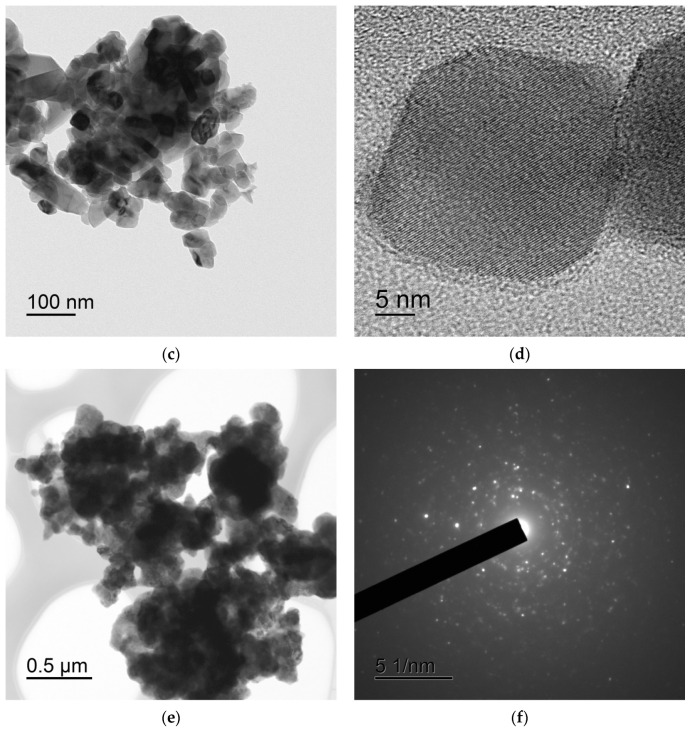
TEM images of the powders Ca_1-x_Zr_2_(PO_4_)_3-x_(MoO_4_)_x_ with x = 0.1 (**a**,**b**), *x* = 0.3 (**c**,**d**), x = 0.4 (**e**,**f**).

**Figure 5:**
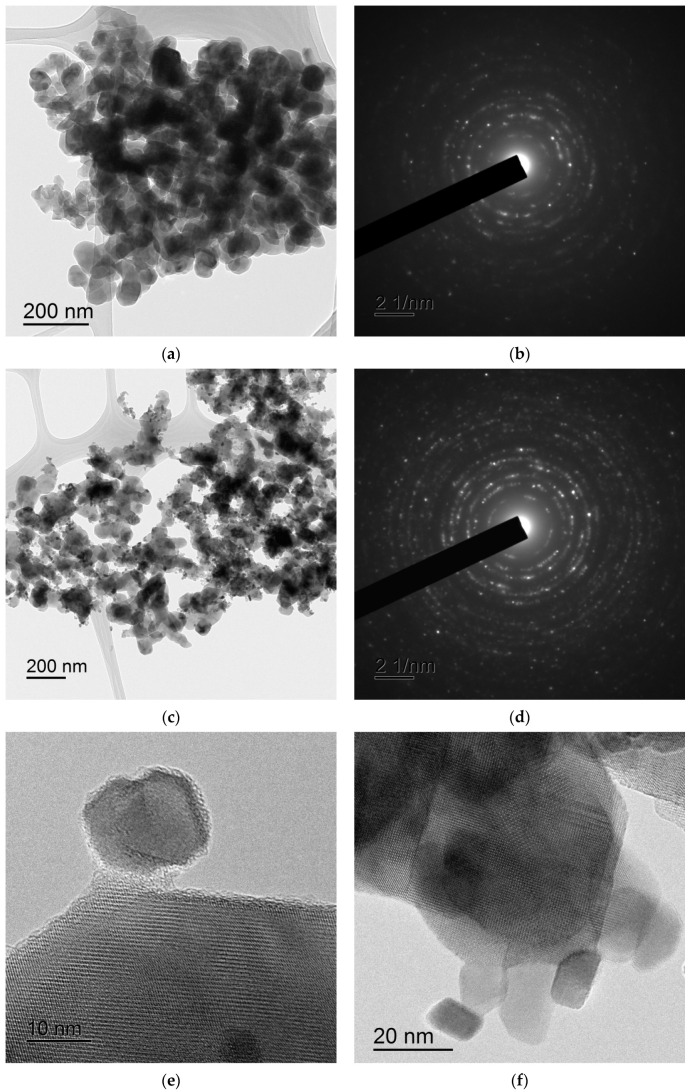
TEM images of the powders Na_0.5_Zr_2_(PO_4_)_2.5_(XO_4_)_0.5_: X = Mo (**a**,**b**,**e**), X = W (**c**,**d**,**f**).

Figure 4 and Figure 5 present TEM images of the Ca_1-x_Zr_2_(PO_4_)_3-x_(MoO_4_)_x_ powders with different Mo contents (Figure 4) and of Na_0.5_Zr_2_(PO_4_)_2.5_(XO_4_)_0.5_ powders, X = Mo, W (Figure 5). The Ca_1-x_Zr_2_(PO_4_)_3-x_(MoO_4_)_x_ powders were large-grained and agglomerated strongly. The individual particle sizes ranged from ~50–100 nm to ~300 nm and didn’t depend on the Mo content (Figure 4). The Na_0.5_Zr_2_(PO_4_)_2.5_(XO_4_)_0.5_ powders were more finely dispersed and agglomerated. The particle sizes ranged from ~20–30 nm to ~50–100 nm (Figure 5). The agglomerate sizes were ~0.2–0.3 μm. All powders had crystalline structure (Figure 4b,d,f and Figure 5b,d,e,f).

**Figure 6:**
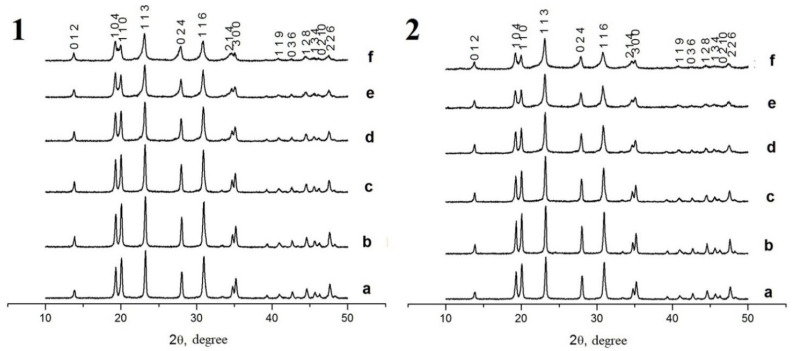
XRD data. Phosphate Na_1-x_Zr_2_(PO_4_)_3-x_(XO_4_)_x_ compounds, X = Mo (**1**), W (**2**), *x* = 0 (a), 0.1 (b), 0.2 (c), 0.3 (d), 0.4 (e), 0.5 (f).

**Figure 7:**
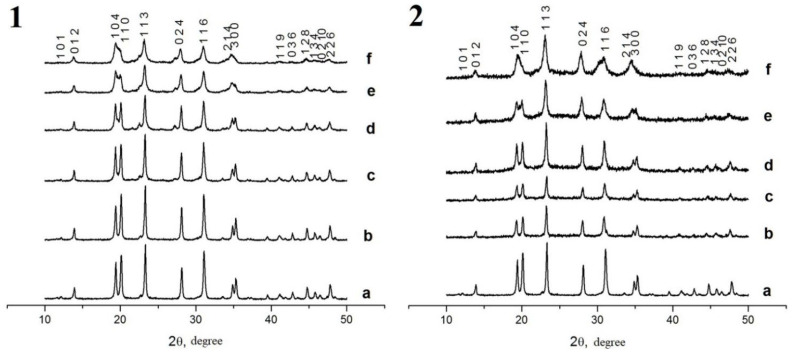
XRD data. Phosphate Ca_1-x_Zr_2_(PO_4_)_3-x_(XO_4_)_x_ compounds, X = Mo (**1**), W (**2**), *x* = 0 (a), 0.1 (b), 0.2 (c), 0.3 (d), 0.4 (e), 0.5 (f).

According to the XRD phase analysis data (Figure 6 and Figure 7), the compounds under study crystallized in the NaZr_2_(PO_4_)_3_ type structure, hexagonal syngony, space group R$\bar{3}$c (analog of NaZr_2_(PO_4_)_3_ [37]) for the Na-containing samples and space group R$\bar{3}$ (analog of Ca_0.5_NaZr_2_(PO_4_)_3_ [68]) for the Ca-containing ones. The XRD patterns of the samples with Ca at x > 0.2 for phosphate–molybdates and x > 0.3 for phosphate–tungstates contained a significant number of auxiliary (secondary) phase reflections. For this reason, these samples were not studied further.

**Figure 8:**
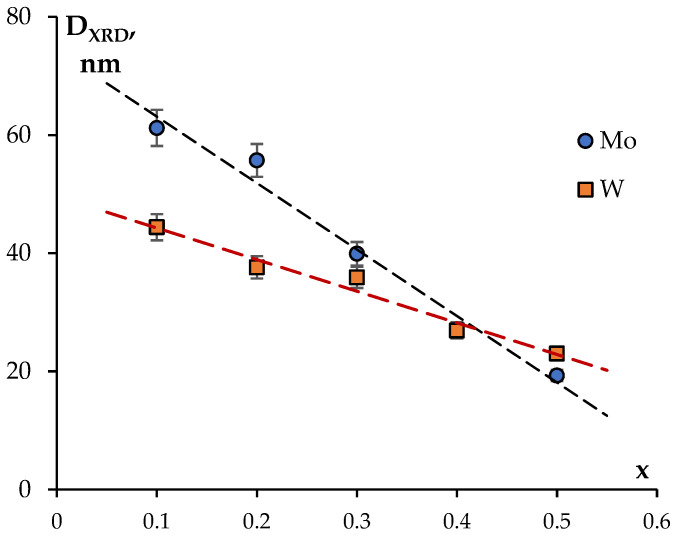
Dependencies of the CSR sizes (D_XRD_) on the Mo and W contents in the Na_1-x_Zr_2_(PO_4_)_3-x_(XO_4_)_x_ powders.

The particle (coherent scattering region, CSR) sizes D_XRD_ were estimated using the Scherer equation:

D_XRD_ = K·λ/β·cosθ_max_, (2)

where K = 0.9 is the Scherer constant, λ is the wavelength of the X-ray radiation, β is the full width at half maximum of the XRD peak (in radians), and θ_max_ is the diffraction angle corresponding to the (116) XRD peak maximum. The analysis of the XRD results demonstrated that the particle sizes D_XRD_ of the Na_1-x_Zr_2_(PO_4_)_3-x_(MoO_4_)_x_ powders decreased monotonously from 61 nm down to 19 nm with increasing Mo content from 0.1 up to 0.5 and from 44 nm down to 23 nm with increasing W content from 0.1 up to 0.5 (Figure 8). No considerable effects of W and Mo content on the D_XRD_ of the Ca_1-x_Zr_2_(PO_4_)_3-x_(XO_4_)_x_ powders were observed; the mean particle sizes were ~60 nm for the W-containing powders and ~47 nm for the Ca_1-x_Zr_2_(PO_4_)_3-x_(MoO_4_)_x_ ones. 

From the analysis of the XRD, TEM, and SEM results comes the conclusion that partial sintering of several nanoparticles into submicron ones takes place in the course of synthesis, at the last stage of high-temperature annealing (800 °C, 20 h). This conclusion was confirmed by comparing the results of investigations of the mean particle sizes by XRD, TEM, and SEM. The analysis of the XRD results shows that the mean CSR sizes in the NZP and CZP compounds were several tens of nanometers (D_XRD_ < 60 nm). The results of TEM and SEM show the particles to be agglomerated strongly; the agglomerates of submicron and micron sizes consist of separate nanoparticles (Figure 2, Figure 3 and Figure 4).

The unit cell parameters for the solid solutions were determined from the XRD data (the results are presented in Table 1).

**Figure 9:**
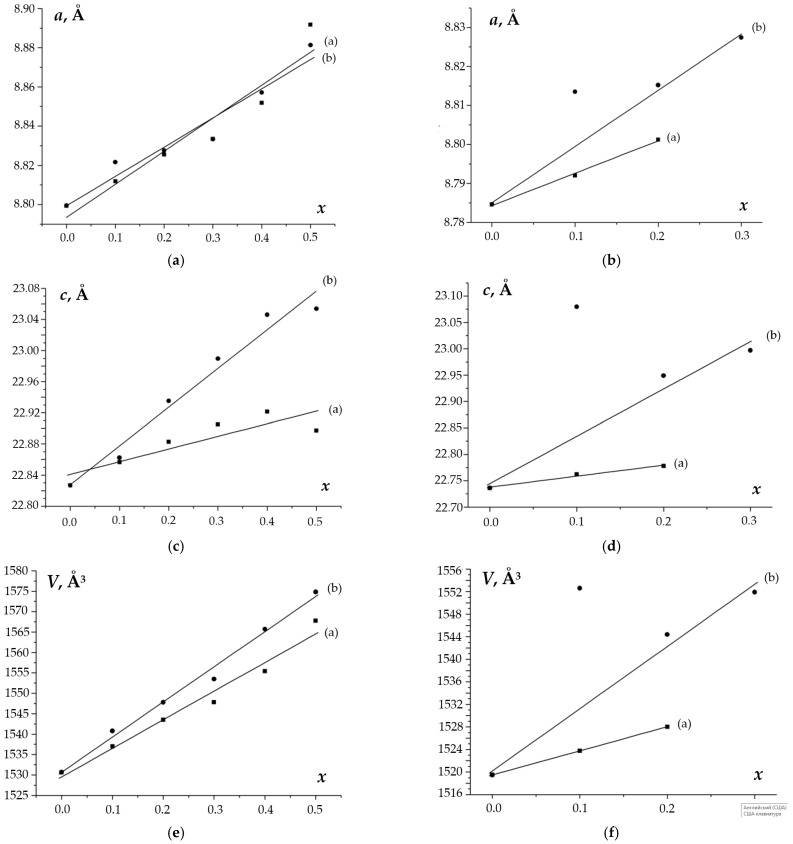
Dependencies of the unit cell parameters *a* and *c*, and of the unit cell volume *V* on the composition (*x*) of phosphates Na_1-x_Zr_2_(PO_4_)_3-x_(XO_4_)_x_ (**a**,**c**,**e**) and Ca_0.5(1-x)_Zr_2_(PO_4_)_3-x_(XO_4_)_x_ (**b**,**d**,**f**), X = Mo (**a**), W (**b**). The linear fit of the data for the Ca_0.5(1-x)_Zr_2_(PO_4_)_3-x_(XO_4_)_x_ compounds was performed without taking into account the data for *x* = 0.1. The reliability coefficient of linear fit for Ca_0.5(1-x)_Zr_2_(PO_4_)_3-x_(XO_4_)_x_ was R^2^ > 0.9 without taking into account *x* = 0.1.

The composition dependences of the unit cell parameters for the phosphate–molybdates and phosphate–tungstates are presented in Figure 9. Substitution of phosphate anions (PO_4_)^3−^ (R (P^5+^) = 0.17 Å) by larger molybdate anions (MoO_4_)^2−^ (R (Mo^6+^) = 0.41 Å) and tungstate ions (WO_4_)^2−^ (R(W^6+^) = 0.44 Å) leads to an increase in the unit cell parameters. One can see from Figure 9 that increasing the W content in the Na_1-x_Zr_2_(PO_4_)_3-x_(XO_4_)_x_ compounds results in a greater increase in the lattice parameter *c* and the unit cell volume *V* than increasing the Mo content. No essential differences in the values of the lattice parameter *a* for the phosphate–molybdates and phosphate–tungstates Na_1-x_Zr_2_(PO_4_)_3-x_(XO_4_)_x_ were observed. For the Ca–phosphate–tungstates Ca_1-x_Zr_2_(PO_4_)_3-x_(WO_4_)_x_, no dependencies of the unit cell parameters *a* and *c*, nor the unit cell volume *V*, on the W content were found. In the case of Ca_1-x_Zr_2_(PO_4_)_3-x_(MoO_4_)_x_, the increase in the Mo content up to x = 0.2 resulted in a linear increase in all unit cell parameters and, hence, in a linear increase in the unit cell volume *V*.

**Figure 10:**
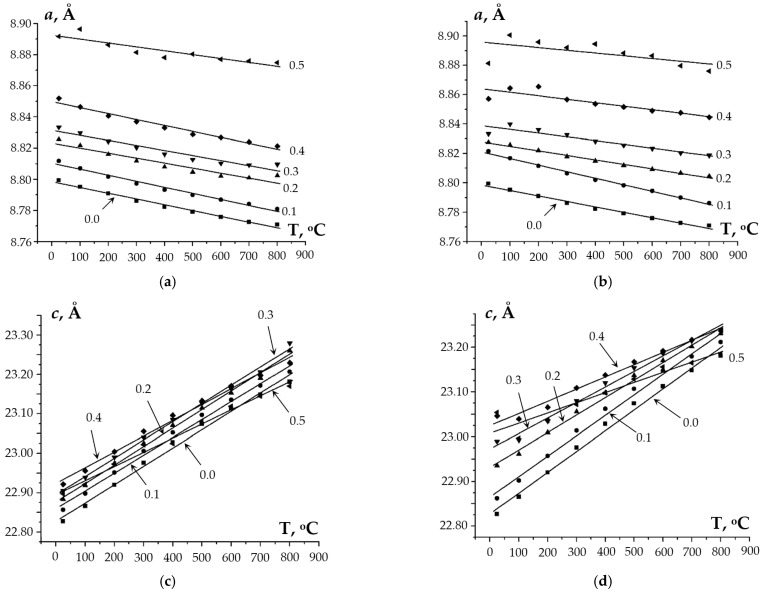

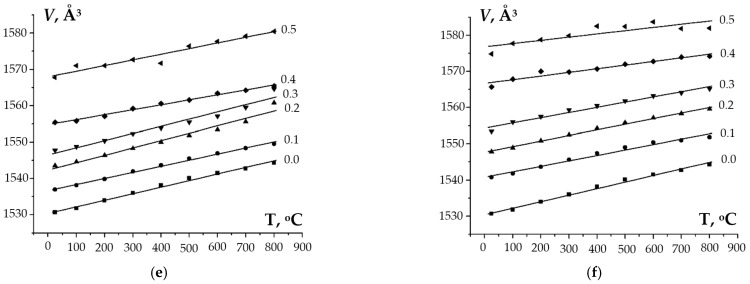
Temperature dependencies of the unit cell parameters *a* and *c*, and of the unit cell volume *V* of phosphates of Na_1-x_Zr_2_(PO_4_)_3-x_(XO_4_)_x_, X = Mo (**a**,**c**,**e**), W (**b**,**d**,**f**); *x* = 0, 0.1, 0.2, 0.3, 0.4, 0.5.

**Figure 11:**
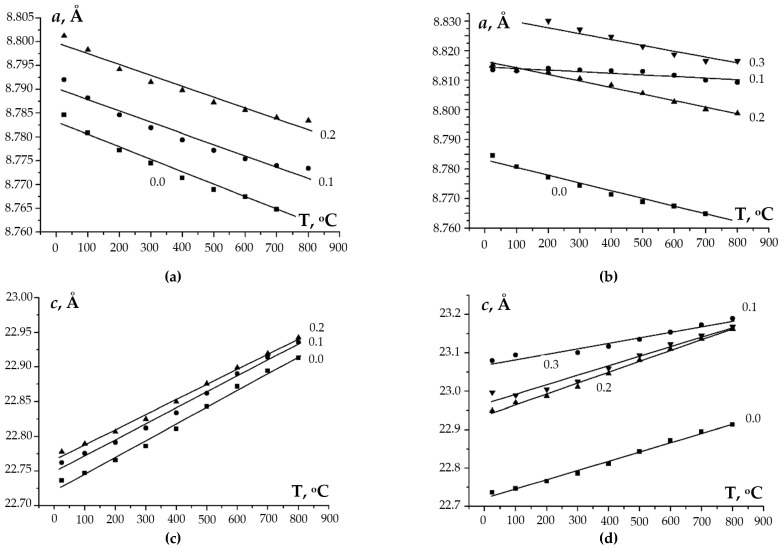

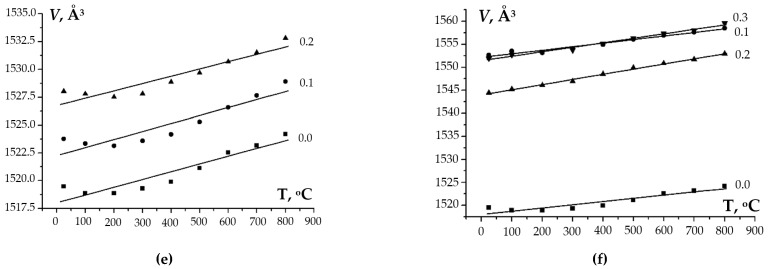
Temperature dependencies of the unit cell parameters *a* and *c*, and of the unit cell volume *V* of phosphates of Ca_1-x_Zr_2_(PO_4_)_3-x_(XO_4_)_x_, X = Mo (**a**,**c**,**e**), W (**b**,**d**,**f**); *x* = 0, 0.1, 0.2, 0.3.

To study the behavior of the obtained compounds upon heating, the XRD patterns of the samples were recorded at elevated temperatures (RT–800 °C). The XRD data were used to calculate the values of the unit cell parameters at different temperatures. The temperature dependencies of the cell parameters are shown in Figure 10 and Figure 11. One can see from Figure 10 a minor decrease (~0.02 Å) in the unit cell parameter *a* and a considerable increase in the parameter *c* (~0.2–0.4 Å) with increasing temperature (from 20 up to 800 °C) for Na_1-x_Zr_2_(PO_4_)_3-x_(XO_4_)_x_. This leads to an increase in the unit cell volume *V* when heating. For Ca_1-x_Zr_2_(PO_4_)_3-x_(XO_4_)_x_, a greater increase in the parameter *c* was observed with a similar increase in temperature (Figure 11), which resulted in a smaller increase in the unit cell volume of Ca_1-x_Zr_2_(PO_4_)_3-x_(XO_4_)_x_ as compared to Na_1-x_Zr_2_(PO_4_)_3-x_(XO_4_)_x_.

**Figure 12:**
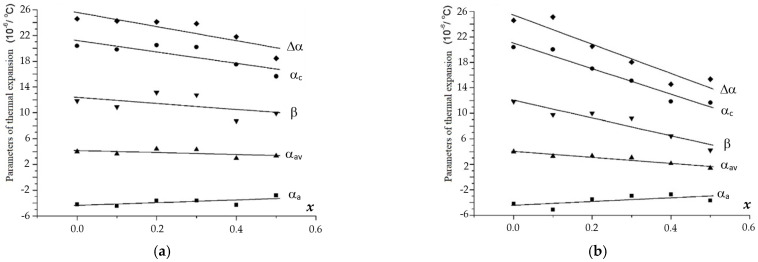
Dependencies of the thermal expansion parameters (α*_a_*, α*_c_*, α_av_, β, and Δα) on the compositions (*x*) of phosphates Na_1-x_Zr_2_(PO_4_)_3-x_(XO_4_)_x_, X = Mo (**a**), W (**b**).

**Figure 13:**
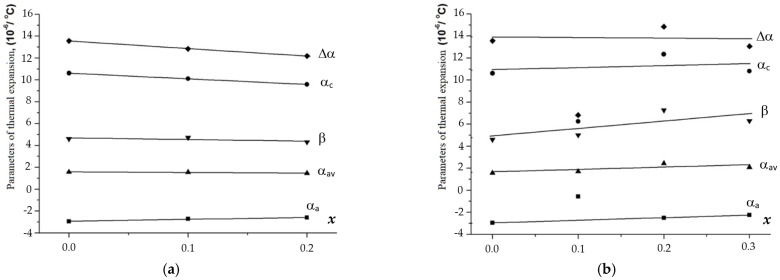
Dependencies of the thermal expansion parameters (α*_a_*, α*_c_*, α_av_, β, and Δα) on the compositions (*x*) of phosphates Ca_0.5(1-x)_Zr_2_(PO_4_)_3-x_(XO_4_)_x_, X = Mo (**a**), W (**b**).

The above dependencies were used to calculate the values of the axial (α_a_ and α_c_), average (α_av_), and volume (β) thermal expansion coefficients (CTEs), as well as the thermal expansion anisotropy (Δα) for the phosphate–molybdates and phosphate–tungstates under study (Table 2, Figure 12 and Figure 13). The analysis of these data shows the decreasing of α_a_ and α_c_ with increasing W and Mo contents in Na_1-x_Zr_2_(PO_4_)_3-x_(XO_4_)_x_. At the same time, the mean value of α_av_ changes slowly. The volume CTE (β) decreases slowly as the thermal expansion anisotropy (Δα) increases. In general, a similar character of the CTE dependencies on the composition was observed for the Ca_1-x_Zr_2_(PO_4_)_3-x_(MoO_4_)_x_ compounds with the increase I n the Mo content (Figure 12). For the W-containing phosphates Ca_1-x_Zr_2_(PO_4_)_3-x_(WO_4_)_x_, no considerable effect of the W content on all thermal expansion parameters (α_a_, α_c_, α_av_, β, and Δα) was observed.

One can note a trend for the thermal expansion parameters to approach zero with decreasing population of the extraframe positions in the structure of the phosphate–molybdates and phosphate–tungstates under study. Therefore, novel ceramic materials with zero CTE and high thermal shock resistance can potentially be developed on the basis of the phosphates with predefined W and/or Mo contents studied in the present work.

### 3.2. Sintering and Characterization of the Ceramics

**Figure 14:**
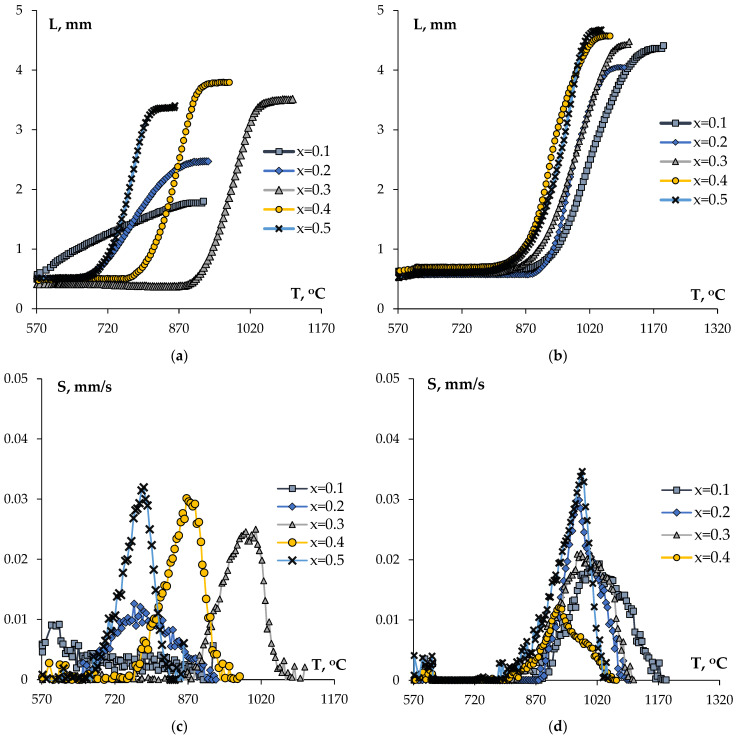
SPS diagrams L(T) (**a**,**b**) and S(T) (**c**,**d**) for the Na_1-x_Zr_2_(PO_4_)_3-x_(XO_4_)_x_ ceramics, X = Mo (**a**,**c**), W (**b**,**d**).

The ceramic samples with high relative densities were obtained from the Na-containing compounds by SPS. The ceramic samples have no visible macro- nor microcracks. Examples of temperature curves L(T) and S(T) are shown in Figure 14. The shapes of the curves L(T) are typical for SPS of the nano- and submicron NZP-type powders [27,51,53].

The analysis of the temperature curves of the shrinkage L(T) for the phosphate–molybdates (Figure 14a,c) shows an increase in the maximum shrinkage L_max_ and of the shrinkage rate S_max_ with increasing Mo content from x = 0.1 up to 0.3. At the same time, an increase in the optimal sintering temperature was observed, which is manifested as the shift of the curves L(T) and S(T) towards higher heating temperatures. With a further increase in the Mo content up to x = 0.5, a shift of the curves L(T) and S(T) towards lower sintering temperatures was observed. In our opinion, such a behavior is due, first of all, to the appearance of some auxiliary phases during sintering, and to the increasing concentration of the auxiliary phases with increasing Mo concentration (see above).

In the Na_1-x_Zr_2_(PO_4_)_3-x_(WO_4_)_x_ phosphates, a minor increase in the shrinkage and a decrease in the optimal sintering temperature corresponding to the completion of the intensive shrinkage stage with increasing W content were observed (Figure 14b). At the same time, an increase in the maximum shrinkage rate of nanopowders S_max_ of more than three times, and a minor shift of the temperatures, during which the maximum shrinkage rates S_max_ were manifested towards higher heating temperatures, were observed (Figure 14d).

**Figure 15:**
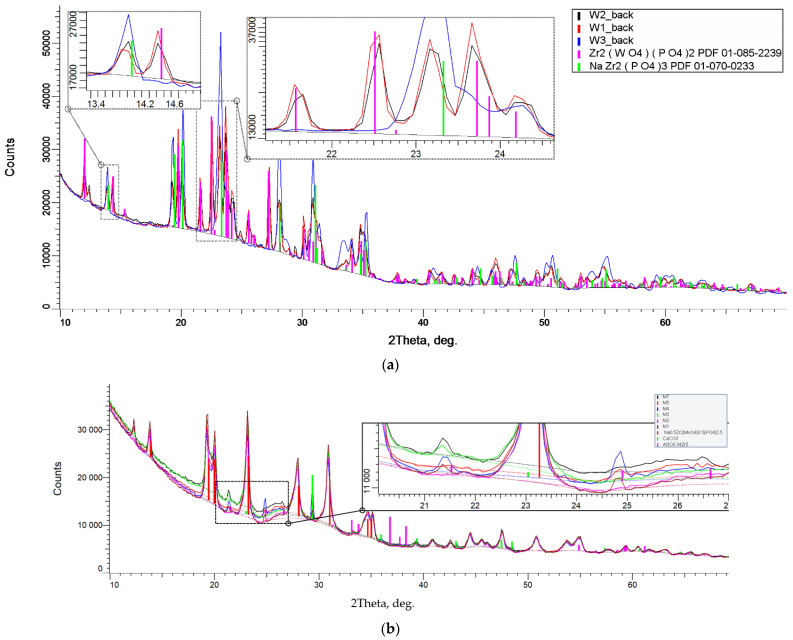
XRD curves of the ceramic samples of phosphate–tungstate Na_1-x_Zr_2_(PO_4_)_3-x_(XO_4_)_x_ ceramics, X = W (**a**), Mo (**b**), with *x* = 0.5: W1–W3—the labels of three phosphate–tungstate satellite ceramic specimens, M1–M7—the labels of seven phosphate-molybdate ones.

The average full SPS process times (t_sps_), including the heating times, were 13 min for the phosphate–molybdates Na_1-x_Zr_2_(PO_4_)_3-x_(MoO_4_)_x_ and 16 min for the phosphate–tungstates Na_1-x_Zr_2_(PO_4_)_3-x_(WO_4_)_x_. The characteristics of the sintering process (the heating rates V_h_, the sintering temperatures T_s_, and the full SPS process time t_sps_), the relative densities achieved, the microhardness values, and the minimal fracture toughness factors for the ceramics obtained are presented in Table 3. One can see from Table 3 that the increasing of the W and Mo contents resulted in an increasing of the relative density of the Na_1-x_Zr_2_(PO_4_)_3-x_(XO_4_)_x_ ceramics. Densities close to the theoretical one were ensured for almost all ceramics. In the ceramics with increased W and Mo contents, the relative densities exceeded 100% slightly. In our opinion, this result originates from the auxiliary (secondary) phases contained in the powders synthesized (see the XRD phase analysis results above). The results of XRD phase analysis of the ceramics also show the secondary phases to be present in the ceramics (Figure 15). In the W-containing ceramics, the presence of Zr_2_(WO_4_)(PO_4_)_2_ secondary phase was observed (Figure 15a), in the Mo-containing ones CaCO_3_ and Al_3_O_0.34_Zr_5_, secondary phases were found (Figure 15b).

One can see from Table 3 that in the NZP ceramics with high Mo and W contents, one can provide a high density at reduced sintering temperatures. We suggest the that an increase in Mo and W contents will result in a decrease in the sintering activation energy for the Na_1-x_Zr_2_(PO_4_)_3-x_(XO_4_)_x_ ceramics, X = Mo, W (0 ≤ *x* ≤ 0.5). This allows sintering the ceramics with high values of *x* up to high densities at lower temperatures. The decrease in the sintering activation energy was manifested indirectly also in a decrease in the temperature at the beginning of the intensive shrinkage stage (Stage II) in Figure 14 at low heating temperatures.

**Figure 16:**
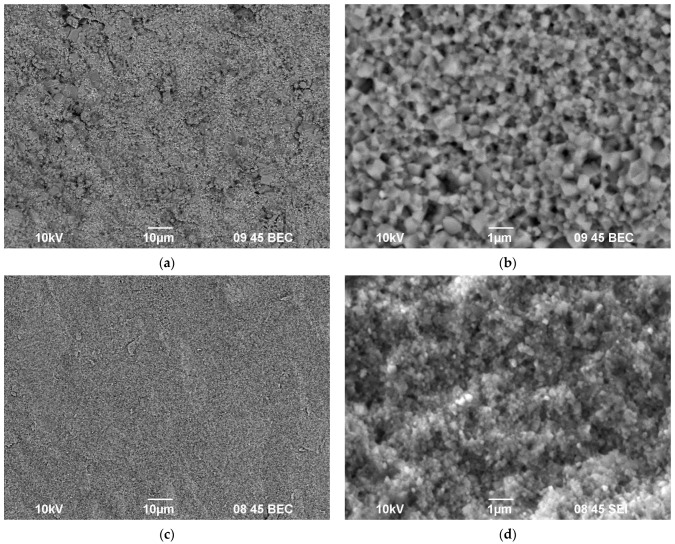
Microstructure of Na_1-x_Zr_2_(PO_4_)_3-x_(MoO_4_)_x_ ceramics with *x* = 0.1 (**a**,**b**) and *x* = 0.5 (**c**,**d**). SEM images of the fractures of sintered ceramic samples (**b**,**d**).

**Figure 17:**
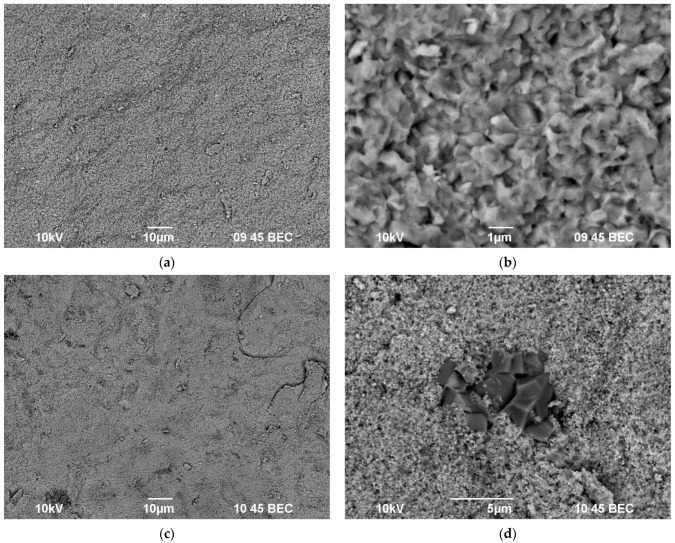
Microstructure of Na_1-x_Zr_2_(PO_4_)_3-x_(WO_4_)_x_ ceramics with *x* = 0.1 (**a**,**b**) and *x* = 0.5 (**c**,**d**). SEM images of the fractures of sintered ceramic samples (**b**,**d**).

Figure 16 and Figure 17 present the SEM images of the microstructure of the sintered phosphate–molybdates Na_1-x_Zr_2_(PO_4_)_3-x_(MoO_4_)_x_ (Figure 16) and phosphate–tungstates Na_1-x_Zr_2_(PO_4_)_3-x_(WO_4_)_x_ (Figure 17) with various Mo and W contents. One can see in Figure 16 a decrease in the mean grain size (Figure 16b,d) and an increase in the microstructure uniformity of the ceramic phosphate–molybdates (Figure 16a,c) with increasing Mo content. Note that there were some areas with abnormally large grains in the sintered ceramics with low Mo contents (x = 0.1). The sizes of these grains were ~5–10 μm (Figure 16a), greater than the mean grain size of the ceramic matrix (Figure 16b) by an order of magnitude. In the ceramics with increased Mo contents (x = 0.5), the areas with abnormally large grains were almost absent (Figure 16c), whereas the mean grain sizes in the sintered ceramic phosphate-molybdates were close to 100–200 nm (Figure 16d).

A similar effect has been observed when analyzing the results of SEM investigations of the ceramic phosphate–tungstates with different W contents. As one can see in Figure 17, an increase in the W content resulted in a decrease in the mean grain sizes in the ceramics down to ~100–150 nm. There are few areas with abnormally large grains, the sizes of which is not greater than 2–5 μm in the ceramics with x = 0.5 (Figure 17d).

The XRD phase analysis evidenced the phase composition of the ceramics not to change after sintering (Figure 15). A minor increase in the intensities and decrease in the widths of the XRD peaks have been observed that is typical for sintering the nano- and submicron powders, which is associated with the grain growth.

As one can see from Table 3, the increase in the Mo and W contents resulted in a minor increase in the microhardness of the ceramics up to 5.3–5.6 GPa. At the same time, the minimum fracture toughness factor almost did not change and exceeded 1 MPa·m^1/2^ as a rule, which is a typical value for the mineral-like ceramics with the NZP structure [4,56].

## 4. Conclusions

1. The submicron-grade powders of the Na_1-x_Zr_2_(PO_4_)_3-x_(XO_4_)_x_ and Ca_1-x_Zr_2_(PO_4_)_3-x_(XO_4_)_x_ compounds, X = Mo, W (0 ≤ x ≤ 0.5) were obtained by sol-gel synthesis. The powders were agglomerated; large agglomerates of several microns in sizes consisted of individual nano- and submicron-sized particles. The sizes of individual particles of the Ca_1-x_Zr_2_(PO_4_)_3-x_(XO_4_)_x_ compounds varied from 50–100 to 300 nm; the ones for the Na_1-x_Zr_2_(PO_4_)_3-x_(XO_4_)_x_ compounds varied from ~20–30 nm to ~50–100 nm.
2. It follows from the analysis of the results obtained that the investigated compounds crystallize in the NZP-type structure, hexagonal syngony, R$\bar{3}$c space group for the Na-containing phosphate–molybdates and phosphate–tungstates and R$\bar{3}$ for the Ca-containing ones. The substitution of the phosphate anions (PO_4_)^3−^ (R(P^5+^) = 0.17 Å) by a larger molybdate (MoO_4_)^2−^ (R (Mo^6+^) = 0.41 Å) and tungstate (WO_4_)^2−^ (R(W^6+^) = 0.44 Å) ions leads to an increasing of the unit cell parameters. With a decrease in the population of the extraframe positions in the phosphate–molybdates and phosphate–tungstates investigated, the thermal expansion parameters tend to approach zero.
3. The dependencies of the crystal lattice constants *a* and *c* as well as of the unit cell volume *V* on the heating temperature were studied by high-temperature X-ray diffraction. The dependencies of the mean thermal expansion coefficient (TEC) (α*_av_*) and of the volume coefficient (β) on the W and Mo contents in NZP and CZP compounds were investigated. The mean values of TEC (α*_av_*) for the Mo-containing NZP and CZP compounds are (2.97–4.41)·10^−6^ °C^−1^ and (1.45–1.55)·10^−6^ °C^−1^, respectively. The mean values of α*_av_* for the W-containing NZP and CZP compounds are (1.41–3.33)·10^−6^ °C^−1^ and (1.70–2.44)·10^−6^ °C^−1^, respectively. The increase in the tungsten content leads to a decrease in α*_av_* for the compounds with the NZP structure. The values of the volume TEC (β) and of the TEC anisotropy (Δα) for the NZP compounds decrease with increasing W content. The effects of Mo and W on the increase in β and Δα for the CZP compounds are insignificant.
4. The bulk of the NZP ceramic specimens obtained by SPS were characterized by high relative densities (ρ_rel_> 97.5%). The full time of the SPS process, including the heating and cooling-down times, is ~11.5–16.5 min for the Mo-containing ceramics and ~15.1–18.1 min for the W-containing ones. The values of microhardness (H_v_) of the ceramics ranged from 3.6 to 5.6 GPa, and the minimal fracture toughness factor (K_IC_) ranged from 0.9 to 1.6 MPa m^1/2^. The ceramics with the NZP structure have fine-grained microstructure; abnormal grain growth was observed in the ceramics with increased W contents. In the NZP ceramics with high Mo and W contents, one can provide high density at reduced sintering temperature.

## Figures and Tables

**Table 1:** Crystallographic characteristics of the phosphates Na_1-x_Zr_2_(PO_4_)_3-x_(XO_4_)_x_ (denoted as “NZP”) and Ca_0.5(1-x)_Zr_2_(PO_4_)_3-x_(XO_4_)_x_ (denoted as “CZP”).

X	x	*a*, Å		*c*, Å		*V*, Å^3^	
		NZP	CZP	NZP	CZP	NZP	CZP
-	0	8.799(4)	8.784(6)	22.826(7)	22.736(0)	1530.6(7)	1519.4(7)
Mo	0.1	8.811(8)	8.792(0)	22.856(6)	22.762(2)	1536.9(9)	1523.7(7)
	0.2	8.825(5)	8.801(2)	22.882(7)	22.777(7)	1543.5(3)	1528.0(2)
	0.3	8.833(4)	-	22.904(9)	-	1547.8(0)	-
	0.4	8.851(9)	-	22.921(5)	-	1555.4(2)	-
	0.5	8.891(7)	-	22.897(1)	-	1567.7(8)	-
W	0.1	8.821(6)	8.813(5)	22.862(2)	23.079(8)	1540.7(7)	1552.6(0)
	0.2	8.827(5)	8.815(2)	22.935(1)	22.949(0)	1547.7(9)	1544.3(9)
	0.3	8.833(3)	8.827(4)	22.989(5)	22.996(8)	1553.4(7)	1551.9(0)
	0.4	8.857(1)	-	23.046(1)	-	1565.7(0)	-
	0.5	8.881(3)	-	23.053(7)	-	1574.8(0)	-

**Table 2:** Thermal expansion coefficients [in °C^−1^] of phosphates Na_1-x_Zr_2_(PO_4_)_3-x_(XO_4_)_x_ (denoted as “NZP”) and Ca_0.5(1-x)_Zr_2_(PO_4_)_3-x_(XO_4_)_x_ (denoted as “CZP”).

X	x	α*_a_*·10^6^		α*_c_*·10^6^		α*_av_*·10^6^		β·10^6^		Δα·10^6^	
		NZP	CZP	NZP	CZP	NZP	CZP	NZP	CZP	NZP	CZP
-	0	−4.20	−2.96	20.37	10.60	3.99	1.56	11.82	4.60	24.56	13.56
Mo	0.1	−4.43	−2.73	19.82	10.10	3.66	1.55	10.90	4.71	24.25	12.83
	0.2	−3.63	−2.61	20.50	9.57	4.41	1.45	13.14	4.30	24.12	12.18
	0.3	−3.62	-	20.20	-	4.32	-	12.75	-	23.82	-
	0.4	−4.29	-	17.49	-	2.97	-	8.75	-	21.79	-
	0.5	−2.81	-	15.64	-	3.34	-	9.92	-	18.45	-
W	0.1	−5.10	−0.57	20.03	6.24	3.28	1.70	9.75	5.00	25.13	6.81
	0.2	−3.51	−2.50	17.00	12.33	3.33	2.44	9.99	7.26	20.52	14.83
	0.3	−2.94	−2.26	15.09	10.80	3.07	2.09	9.23	6.29	18.04	13.06
	0.4	−2.71	-	11.85	-	2.14	-	6.45	-	14.56	-
	0.5	−3.71	-	11.65	-	1.41	-	4.23	-	15.36	-

**Table 3:** Characteristics of ceramic samples of phosphates Na_1-x_Zr_2_(PO_4_)_3-x_(XO_4_)_x_.

X	x	V_h_, ^o^C/min	T_s_, °C	t_sps_, min	ρ, %	H_v_, GPa	K_IC_, MPa·m^1/2^
Mo	0.1	50	920	12.58	97.55	3.6 ± 0.5	1.0 ± 0.3
	0.2		932	13.08	98.42	4.5 ± 0.8	1.1 ± 0.5
	0.3		1110	16.50	100.07	5.3 ± 0.5	0.9 ± 0.3
	0.4		975	13.58	100.06	5.1 ± 0.8	1.1 ± 0.6
	0.5		861	11.50	101.66	5.3 ± 0.5	1.4 ± 0.3
W	0.1	50	1190	18.08	97.70	5.3 ± 0.5	1.3 ± 0.3
	0.2		1100	16.83	98.75	5.6 ± 0.6	1.6 ± 0.3
	0.3		1110	17.58	98.59	5.6 ± 0.5	1.5 ± 0.3
	0.4		1065	15.67	100.20	5.5 ± 0.4	1.1 ± 0.3
	0.5		1065	15.08	100.70	5.6 ± 0.6	1.1 ± 0.6

## Data Availability

Not applicable.
